# Fevers—Congestive and Inflammatory

**Published:** 1887-02

**Authors:** C. Coteman Benson


### FEVERS, (CONGESTIVE AND INFLAMMATORY.)

BY C. COTEMAN BENSON, M. D.

All the animal, earthy and chemical properties which constitute the elements of the body act on each other in every form of combination, having a constant tendency to their complex reproduction. Those elements which are unfit to be retained, and which are always of an *acid* character and known as excrementitions, are removed from the body as in excess of its requirements. Life then, as well as all the bodily organs, being dependent on chemical decomposition and recomposition, is a continuous conflict between acids and alkalies in all their combinations. The acids maintain, not only the chemical but the electrical integrity of the system, and must therefore be slightly dominant to induce and preserve the vital and healthful balance. But if this acid dominance over the alkaline elements become much in excess, then the first or congestive state is established, and congestion of the nervous system and mucous membrane is the result, producing congestive fever ; shown by the broad flabby, thickly-coated *white* tongue, increased mucous secretion from the stomach, bad taste in the mouth, weight and heaviness in epigastrium with nausea or vomiting, rapid pulse and heightened temperature.

If this state continues and nature's efforts—as emisis or diarrhoea cannot relieve it, then these poisonous elements enter the arterial current and an inflammatory state is superadded to a congestive one, known as inflammatory fever, indicated by a bright red tongue, elongated and pointed, with erect and reddened papillae, constriction and pain on pressure over epigastrium, with nausea, vomiting, &c.

In the congestive fevers, the mucous membranes only are involved, and functional action is disturbed but not interrupted. For want of moisture, from suppressed secretion, these membranes become inflamed, and we are unable to foresee where this irritation will locate itself, whether in the lungs as bronchitis, in the stomach as gastritis, or in the intestinal canal as entritis or dysentery.

In the inflammatory fever which always succeeds the congestive one, and can never precede it; nature pursues a law of gradation in all diseases, as in health, through the electrical action. This exhaustion of the acid elements, is carried on with the structural elements only, and the serous membranes become involved by loss of their moist secretions, and then death, ushered in by law—ends the suffering.

There is a marked similarity between the symptoms of fever and those of paralysis of the sympathetic system of nerves due to irritation by poisoned blood in fevers ; for if we divide a sympathetic nerve from its invested organ we observe dilitation of the arterioles, lowered blood pressure, increased circulation, disordered glandular secretions and heightened temperature. It is general practice to administer upon these symptoms, aconite, veratrum, antimony, etc., which in very small doses frequently repeated, stimulate the sympathetic ganglia, but in medicinal doses produce the very symptoms for the removal of which they were given, and often paralyze these ganglia even to a fatal issue. The pathology of fever, however, demands different treatment ; for if congestive fever is due to an excess of acid in the system which is its basic lesion, then alkalies with free elimination of the morbid blood elements should be the plan adopted ; while in the inflammatory fever which is due to deficiency of acid elements in the system, acids with anodynes should be given, combined with the most careful avoidance of any attempt at either cathartics or other excretory effort.—*St. Louis Med. Journal.*

